# STING contributes to lipopolysaccharide-induced tubular cell inflammation and pyroptosis by activating endoplasmic reticulum stress in acute kidney injury

**DOI:** 10.1038/s41419-024-06600-1

**Published:** 2024-03-14

**Authors:** Yun Cao, Xinghua Chen, Zijing Zhu, Zilv Luo, Yiqun Hao, Xueyan Yang, Jun Feng, Zongwei Zhang, Jijia Hu, Yonghong Jian, Jiefu Zhu, Wei Liang, Zhaowei Chen

**Affiliations:** 1 Division of Nephrology, Renmin Hospital of Wuhan University, Wuhan, China; 2 Department of Nephrology, Hainan General Hospital (Hainan Affiliated Hospital of Hainan Medical College), Haikou, China; 3 Department of Organ Transplantation, Renmin Hospital of Wuhan University, Wuhan, China

**Keywords:** NOD-like receptors, Apoptosis

## Abstract

Recently, innate immunity and inflammation were recognized as the key factors for acute kidney injury (AKI) caused by sepsis, which is closely related to high mortality. Stimulator of interferon genes (STING) has emerged as a critical component of innate immune and inflammatory responses. However, the role of STING in the pathogenesis of septic AKI remains unclear. This study demonstrated that the STING was significantly activated in tubular cells induced by lipopolysaccharide (LPS) in vivo and in vitro. Tubule-specific STING knockout attenuated LPS-induced renal dysfunction and pathological changes. Mechanistically, the STING pathway promotes NOD-like receptor protein 3 (NLRP3) activation. STING triggers endoplasmic reticulum (ER) stress to induce mitochondrial reactive oxygen species (mtROS) overproduction, enhancing thioredoxin-interacting protein activation and association with NLRP3. Eventually, the NLRP3 inflammasome leads to tubular cell inflammation and pyroptosis. This study revealed the STING-regulated network and further identified the STING/ER stress/mtROS/NLRP3 inflammasome axis as an emerging pathway contributing to tubular damage in LPS-induced AKI. Hence, targeting STING may be a promising therapeutic strategy for preventing septic AKI.

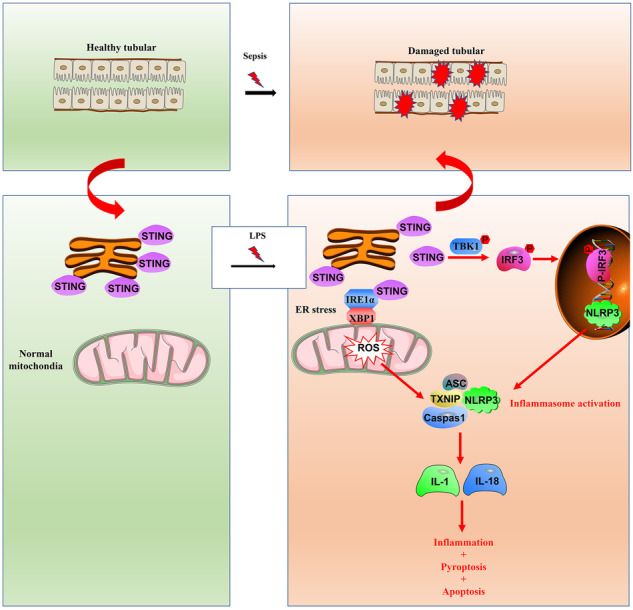

## Introduction

Acute kidney injury (AKI), manifested as a rapid decline in renal function, has become a global health concern [1]. As the leading cause of AKI in hospitalized and critically ill patients, sepsis is characterized by inappropriate immune responses, overwhelming inflammation and multiple organ dysfunction [2]. Unfortunately, septic AKI currently lacks targeted drug treatments and is associated with a high mortality rate. The pathogenesis of septic AKI involves several renal cell types and factors. Among these, the immune and inflammatory response-induced injury and loss of tubular epithelial cells are recognized as the direct and major factors [3, 4]. Therefore, exploring the key mechanism underlying sepsis-induced tubular immune and inflammatory responses is necessary to find novel therapeutic strategies to prevent septic AKI.

Innate immunity, a necessary process for protective anti-infectious immune responses, has become a critical factor of the heightened inflammatory immune state [5]. In sepsis, innate immune responses are triggered by specific pattern recognition receptors (PRRs) by pathogen- or danger-associated molecular patterns [6]. Afterward, the enhanced secretion of inflammatory cytokines amplifies local tissue inflammation, leading to organ dysfunction. Stimulator of interferon genes (STING), a pivotal PRR, physically resides on the surfaces of the endoplasmic reticulum (ER) and outer mitochondrial membranes, which are generally defined as mitochondria-associated membranes (MAMs). Under stress conditions, STING translocates to the ER–Golgi intermediate compartment and Golgi for interaction with TANK-binding kinase 1 (TBK1). The STING-TBK1 complexes then activate nuclear factor (NF)-κB and interferon regulatory factor 3 (IRF3) to promote the transcription of type I interferon (IFN) and other proinflammatory cytokines [7, 8]. Increasing studies have indicated that STING plays an essential role in kidney disease. For instance, limiting STING activity ameliorates renal fibrosis [9]. Aberrant activation of the STING signalosome was observed in ischemia-reperfusion injury and cisplatin-induced AKI, while STING global knockout or STING antagonist H151 pretreatment attenuated renal dysfunction and tubular inflammatory damage [10–12]. However, the potential effects and precise mechanisms of STING in septic AKI (the AKI most closely related to innate immunity) have not been well investigated until now.

Emerging studies have reported that innate immune and inflammatory responses are modulated by ER stress and oxidative stress, and then take effect through the NOD-like receptor protein 3 (NLRP3) inflammasome [13, 14]. Furthermore, ER stress, oxidative stress and the NLRP3 inflammasome were widely regarded as critical pathophysiological pathways in septic AKI, especially related to the sepsis-activated STING pathway [15–17]. In cecal ligation and puncture-induced sepsis, ER stress was markedly blocked by STING deletion [18]. In lipopolysaccharide (LPS)-induced acute lung injury, oxidative stress and NLRP3 inflammasome mediated-pyroptosis (a form of programmed inflammatory cell death) were relieved by STING deficiency [19]. Unfolded protein response (UPR), the most common form of ER stress, is activated by three sensors: inositol requiring protein1 (IRE1), protein kinase RNA-like ER kinase (PERK) and activating transcription factor 6 (ATF6) [20]. The NLRP3 inflammasome, the most-studied inflammasome in generating damaging inflammation, is assembled by NLRP3, thioredoxin-interacting protein (TXNIP), apoptosis speck-like protein (ASC) and pro-caspase1 (casp1). Casp1 cleaves pro-Interleukin-1β/18 (pro-IL-1β/18, pro-inflammatory factors) and gasdermin D (GSDMD, pyroptosis inducer) to initiate inflammation and pyroptosis [21, 22]. Interestingly, ER stress appears to activate the NLRP3 inflammasome by promoting oxidative stress. Salvianolic acid B suppresses NLRP3 activation during ER stress and mitochondrial reactive oxygen species (mtROS) accumulation to prevent endothelial injury. Activating IRE1α increases mtROS level, which promotes NLRP3 association with mitochondria [23]. However, in septic AKI, whether the STING pathway lies upstream and is responsible for the three pathophysiological pathways remains unclear.

The endotoxin LPS is a potent trigger of host defense and the most common cause of sepsis. Here, we explored the role of STING in LPS-induced AKI. Then, whether STING contributes to tubular cell injury by activating ER stress, oxidative stress and the NLRP3 inflammasome was studied, as well as the potential molecular mechanisms among them. This study aimed to find a novel therapeutic target for septic AKI.

## Materials and methods

### Animals

Male C57BL/6J mice (6–8 weeks old, weighting 20–25 g) were purchased from Beijing HFK Bioscience Co., Ltd. The mice were randomly divided into three groups, each with six mice: control (CTL), LPS 24 h and LPS 48 h. The septic AKI mice models were induced by intraperitoneal (i.p.) injection of LPS (10 mg/kg of body weight, Sigma-Aldrich, USA). The CTL mice were injected with i.p. an equal volume of 0.9% saline.

The STING exon 3, 4, 5-floxed (STING^fl/fl^) mice (C57BL/6J, stock#031670, Jackson Lab) and Ksp-cadherin-Cre (Ksp-Cre) mice (C57BL/6J, stock#012237, Jackson Lab) were crossed to generate tubule-specific STING knockout (STING^fl/fl^, Cre, STING-cKO) mice. Littermates with homozygous floxed mice without Cre expression were considered wild-type (WT) mice and used as controls. Male WT mice and STING-cKO mice (8 weeks old, weighting 24–26 g) were randomly assigned to receive 0.9% saline or LPS injection (24 h), then divided into four groups, each with six mice: WT, WT + LPS, STING-cKO and STING-cKO + LPS.

Blood samples were collected for serum creatinine (Scr), blood urea nitrogen (BUN) and neutrophil gelatinase-associated lipocalin (NGAL) measurement. 24 h urine samples were collected for albumin-to-creatinine ratios (ACR), kidney injury molecule-1 (KIM-1) and NGAL measurement. All mice were euthanized via pentobarbital sodium (200 mg/kg) i.p. injection and sacrificed humanely after 24 h or 48 h from LPS injection. Kidney samples were collected for further testing. All animals were maintained in the Specific Pathogen-Free conditions (Animal Center of the Renmin Hospital of Wuhan University), humidity 40-70%, temperature 20-25 °C, under 12-hour light/12-hour dark pattern with free access to water and food. The entire experimental process was carried out in a blinded manner. All animal experiments in this study were approved by the Ethical Committee for the Experimental Use of Animals of the Renmin Hospital of Wuhan University, China (WDRM-20221004E).

### Cell culture and treatment

The human proximal tubule cell line (HK2) was purchased from Procell Life Science & Technology Co., Ltd and cultured in DMEM/F12 medium (HyClone, USA) supplemented with 10% fetal bovine serum (FBS, Corning, USA) at 37 °C. The differentiated HK2 cells (70–80% density) were cultured using different mediums: (1) LPS time gradients (0, 6, 12, 24 h) at 5 μg/ml; (2) LPS concentration gradients (0, 1, 5, 10 μg/ml) for 12 h; (3) LPS (−) = without LPS; LPS (+) = 5 μg/ml LPS for 12 h. Cells and cell climbing films were harvested for subsequent testing.

### Cell transfection

Overexpression plasmid transfection was conducted using the X-tremeGENE HP DNA Transfection Reagent (Roche, Swit). Small interfering RNA (siRNA) transfection was conducted using HiPerFect Transfection Reagent (Qiagen, Germany). The differentiated HK2 cells (50–60% density) were transfected with IRE1, NLRP3, STING overexpression plasmid (OE-IRE1, OE-NLRP3, OE-STING, GenePharma Co., Ltd, China) or STING siRNA (si-STING, Sangon, China) to overexpress or knock down target genes. The pcDNA3.1 plasmid (OE-Con, GenePharma Co., Ltd, China) and scrambled-siRNA (si-Con, Sangon, China) were transfected as controls. The corresponding siRNA and overexpression plasmid were transfected into HK2 cells at 24-h intervals. The corresponding HK2 cells were treated with LPS (5 μg/ml, 12 h, Solarbio, China) 36 h after transfection. MitoQ (100 nm, MCE, USA) was added to pretreat the corresponding HK2 cells for 2 h before transfection. Cells and cell climbing films were harvested for subsequent testing.

### Histological analysis

Briefly, fixed renal tissues from mice were dehydrated and paraffin-embedded to prepare renal sections. Afterwards, the renal paraffin sections were deparaffinized, hydrated and antigen-retrieved, then stained with hematoxylin-eosin (HE) and periodic acid-Schiff (PAS) reagents to observe the histopathological changes of renal tubules. The percentage of tubule damage was evaluated by HE staining, which showed tubular dilation/flattening, brush border loss, tubular cast formation and tubular degeneration/vacuolization [24]. The tubular injury score was graded according to the proportion of injured tubules in PAS staining as follows: 0, 1, 2, 3, and 4 corresponding to none, <25%, 25–50%, 50–75%, and 75% [10]. An average percentage was calculated by 10–20 randomly chosen fields (×800) from each mouse.

The apoptosis of tubular cells was detected using a TdT-mediated dUTP nick end labeling (TUNEL) kit (Roche, Swit). Each mouse was selected for 10–20 randomly chosen fields (×800) to count TUNEL-positive cells/field, which quantified the average. All microscopic images were observed using an upright microscope (Olympus, Japan).

### Western blot analysis

The total protein of the renal cortices from mice or HK2 cells was extracted with RIPA buffer (Beyotime, China) containing a 1% protease inhibitor cocktail (Sigma-Aldrich, USA), phosphatase inhibitor phosSTOP™ (one tablet dissolved per 10 ml RIPA buffer; Sigma-Aldrich, USA), and 1% PMSF (Beyotime, China). The supernatants were collected after centrifugation at 13,000 rpm for 5 min (4 °C), then mixed with 20% loading buffer (Servicebio, China) and boiled at 100 °C for 10 min. An equal amount of protein was separated by 8–12% SDS-PAGE (Beyotime, China) and transferred onto PVDF membranes (Millipore, USA). The membranes were blocked with 5% skim milk for 1 h and incubated with primary antibodies overnight at 4 °C. The primary antibodies used are listed in (Table S1). After incubation with horseradish peroxidase (HRP)-conjugated secondary antibodies (Antgene, China) at room temperature for 1 h, the membranes were soaked with enhanced chemiluminescence solution (Servicebio, China) for blot detection. All blots were screened and quantified with an X-ray machine (Bio-Rad, USA).

### Immunohistochemistry (IHC)

The renal paraffin sections from mice were deparaffinized, hydrated, antigen-retrieved, and blocked with 5% bovine serum albumin (BSA, Antgene, China) at room temperature for 1 h, followed by incubation with the primary antibodies overnight at 4 °C. The primary antibodies used are listed in **(**Table S2**)**. After incubation with HRP-conjugated secondary antibodies for 1 h, sections were subjected to DAB solution (containing 0.01% H_2_O_2_) for staining.

Each mouse was selected for 10–20 randomly chosen fields (×800) to count lymphocyte antigen 6 complex locus G6D (Ly6g)- or STING-positive cells/field, which quantified the average. All microscopic images were observed using an upright microscope (Olympus, Japan).

### Immunofluorescence (IF) assay

The renal paraffin sections of mice were pretreated as same as IHC. The cell-climbing films were fixed and then blocked. The primary antibodies used are listed in (Table S3). The Alex Fluor 488/594 Donkey anti-Rabbit/Mouse IgG (H&L) (Antgene, China) were used as secondary antibodies. LTL (FL1321, 1:200, Lifespan, China) is a proximal renal tubule marker. DAPI (ANT063, Antgene, China) is a nucleus marker.

Each mouse was selected for 10–20 randomly chosen fields (×600) to count epidermal growth factor module-containing mucin-like receptor (EMR1, also known as F4/80)-or STING-positive cells/field, which quantified the average. All microscopic images were observed using a confocal microscope (Hitachi, Japan).

### mtROS generation, mitochondrial membrane potential (MMP), and adenosine triphosphate (ATP) production analyses

mtROS generation was measured using the DCFH-DA fluorescent probe (Beyotime, China). The fluorescence of DCF (green) represents the intracellular ROS level. One of the indicators of early apoptosis and mitochondrial function, MMP, was evaluated using a mitochondrial membrane potential assay kit with JC-1 (Beyotime, China). At the low MMP, JC-1 is a monomer that shows green fluorescence. At the high MMP, JC-1 aggregates in the mitochondrial matrix to form the polymer, which displays red fluorescence. The proportion of mitochondrial depolarization, a process in which the MMP reduces and the potential difference between inside and outside mitochondria decreases or disappears, can be measured by the relative ratio of JC-1 red/green fluorescence. All microscopic images were observed using an inverted microscope (Olympus, Japan). ImageJ was used to measure fluorescence intensity and the density per area unit from 10–20 randomly chosen fields of each group. ATP production was measured using ATP Assay Kit (Beyotime, China) and quantified by a microplate reader.

### Flow cytometry

The apoptotic rate of HK2 cells was measured using Annexin V-PE Apoptosis Detection Kit I (BD Pharmingen, USA) and quantified by a Flow Cytometer™ system.

### Quantitative real-time PCR

The total RNA was isolated from the frozen renal cortices of mice or HK2 cells using TRIzol reagent (Invitrogen, USA). cDNA was then synthesized using PrimeScript RT Reagent Kit (Takara, Japan). The target genes (tumor necrosis factor α, TNFα; monocyte chemotactic protein-1, MCP-1; IL-6; granulocyte-macrophage colony stimulating factor, GM-CSF) were amplified (SYBR Green Kit, Takara, Japan) and quantified in a real-time fluorescence-based quantitative PCR machine (Illumina Eco, USA). The sequences of the primers used in this study are listed in (Table S4). The relative gene expression was quantified by the 2^−△△CT^ method, and glyceraldehyde-3-phosphate dehydrogenase (GAPDH) was used as a control for normalization.

### Bulk RNA-sequencing (RNA-seq) analysis

The fresh renal cortices of WT, STING-cKO, WT + LPS and STING-cKO + LPS mice were immersed in TRIZOL buffer, quick-frozen in liquid nitrogen, and then sent to (Novogene Co., Ltd, China) in dry ice packaging for RNA-seq analysis, and the resulting data were deposited in the SRA database (PRJNA1065405). differentially expressed genes (DEGs) were identified with a threshold of Log2 fold-change (L2FC) > 1 or <−1 and an adjusted p < 0.05 to generate heatmaps using TB tolls. Gene ontology (GO) analysis was performed using the Metascape website (https://metascape.org).

### Statistical analyses

All experiments were repeated at least thrice. All data were analyzed using GraphPad Prism7 (GraphPad Software, Inc.). Data were summarized as means ± standard deviation (SD) of the indicated number of independent experiments. One-way analysis of variance (ANOVA) followed by a Tukey’s post-test was applied for statistical comparisons between groups. Statistical significance was regarded as p < 0.05.

## Results

### Activation of the STING/TBK1/IRF3 pathway in LPS-treated tubular cells

**Fig. 1:**
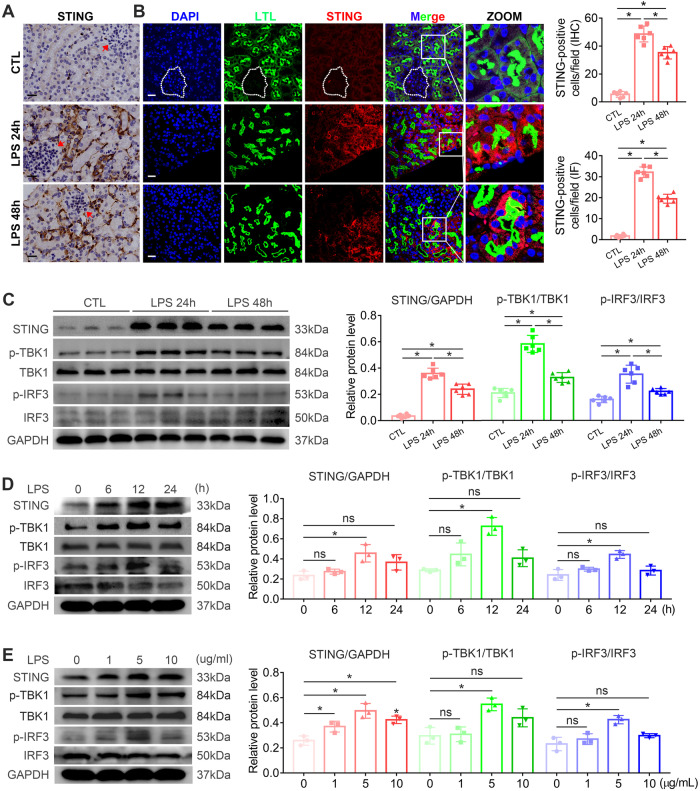
Activation of the STING/TBK1/IRF3 pathway in HK2 cells and mice induced by LPS. **A** Representative images and STING-positive cells quantitation of immunohistochemical staining of STING in renal tubules from mice i.p.-injected with LPS (10 mg/kg) for 0, 24, 48 h (original magnification, ×800). Scale bars: 20μm. Red arrows indicate glomerulus. **B** Representative images and STING-positive cells quantitation of immunofluorescence of STING, LTL (proximal tubule marker) and DAPI (DNA marker) in renal tubules from mice i.p.-injected with LPS (10 mg/kg) for 0, 24, 48 h (original magnification, ×600). Scale bars: 20μm. White outlines indicate glomerulus. **C** Representative western blots and quantitation of STING, p-TBK1, p-IRF3 protein expression in renal cortices from mice i.p.-injected with LPS (10 mg/kg) for 0, 24, 48 h. **A**–**C** *n* = 6 per group. **D**, **E** Representative western blots and quantitation of STING, p-TBK1, p-IRF3 protein expression in HK2 cells treated with LPS time gradients (0, 6, 12, 24 h) for 5 μg/ml and LPS concentration gradients (0, 1, 5, 10 μg/ml) at 12 h. *P < 0.05 vs. indicated group. ns no significant.

An LPS-induced AKI mouse model was established. Interestingly, compared to 24 h, renal pathological improvements were detected after LPS treatment 48 h (Fig. S1A, B). To investigate the alterations of the STING/TBK1/IRF3 pathway in vivo, we observed the intrarenal location and alteration of the STING protein by IHC and IF analyses. Notably, compared to the glomerulus, renal tubules from LPS-induced AKI mice showed more evident STING expression increases (Fig. 1A, B). As indicated in (Fig. 1C), STING, TBK1 and IRF3 were activated in kidney cortices from LPS-induced AKI mice, and the intervention time of LPS in mice was most appropriate when set at 24 h. We next set the time and concentration gradients to detect the activation of the STING/TBK1/IRF3 pathway in LPS-treated HK2 cells. The protein levels of STING, p-TBK1 and p-IRF3 were significantly upregulated with LPS treatment, and mainly peaked at 5 μg/ml, 12 h in vitro (Fig. 1D, E). These results indicated that the activation of the STING/TBK1/IRF3 pathway is involved in the development of tubular damage in LPS-induced AKI.

### STING knockout attenuated tubular damage in LPS-induced AKI

**Fig. 2:**
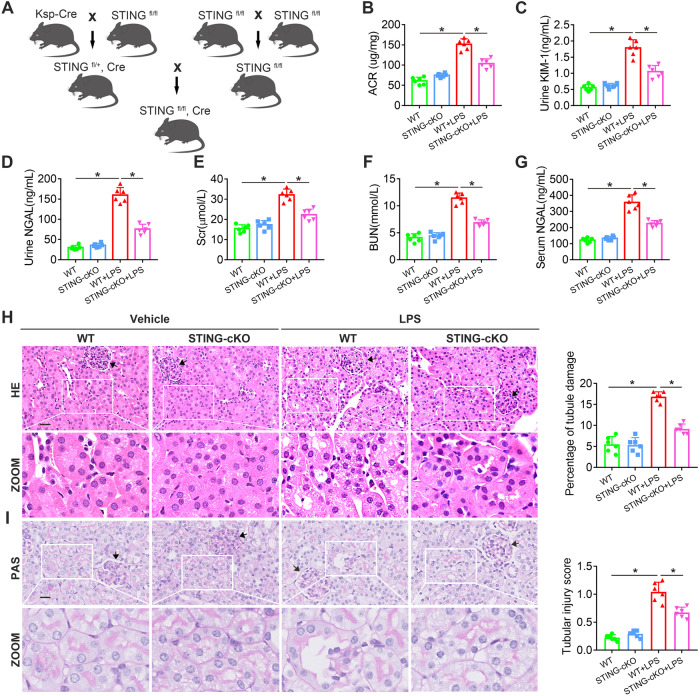
STING-cKO attenuated clinical and histopathological changes in LPS-induced AKI. **A** Strategy for generating STING-cKO mice. **B**–**G** Representative quantitation of ACR, KIM-1, NGAL in urine samples and Scr, BUN, NGAL in blood samples from mice per group. **H**, **I** Representative images of HE and PAS staining of renal tubules from mice per group (original magnification, ×800). Scale bars: 20 μm. Black arrows indicate glomerulus. Representative quantitation of percentage of tubule damage and tubular injury score from mice per group was evaluated by HE and PAS staining. **B**–**I** *n* = 6 per group, *P < 0.05 vs. indicated group.

To further gain insight into the necessity of STING in the tubular damage of LPS-induced AKI, we deleted the STING in renal tubules by crossing Ksp-Cre and STING^fl/fl^ mice (Fig. 2A and Fig. S2A). PCR analysis showed the genotype of STING-cKO (STING^fl/fl^, Cre) mice (Fig. S2B, lane 4) and WT mice (STING^fl/fl^) (Fig. S2B, lane 2). Compared to WT mice, STING-cKO mice did not elicit noticeable changes in renal histopathology (Fig. S2C, D) and had no significant differences in body weight, kidney weight, kidney/body weight ratio (Fig. S2E–G), indicating that STING-cKO mice had no overt phenotype without stress.

Subsequently, we compared the disease progression of WT mice and STING-cKO mice in the model of LPS-induced AKI. LPS treatment rapidly increased urinary ACR, KIM-1, NGAL levels and Scr, BUN, serum NGAL levels in WT mice, indicating renal dysfunction and tubular injury (Fig. 2B–G). HE and PAS staining also showed tubular pathological changes in the WT + LPS group, such as tubular lumen dilation, epithelial vacuolation/flatness, epithelial nucleus exposure and brush border loss (Fig. 2H, I). However, the STING-cKO + LPS group exhibited less affected functional (Fig. 2B–G) and histological (Fig. 2H, I) analyses than the WT + LPS group, demonstrating the protective effect of STING knockout on LPS-induced tubular damage. The above results suggested that STING was an essential pathogenic factor for tubular damage in LPS-induced AKI.

### Effects of STING knockout on LPS-induced tubular inflammation and NLRP3 inflammasome activation

**Fig. 3:**
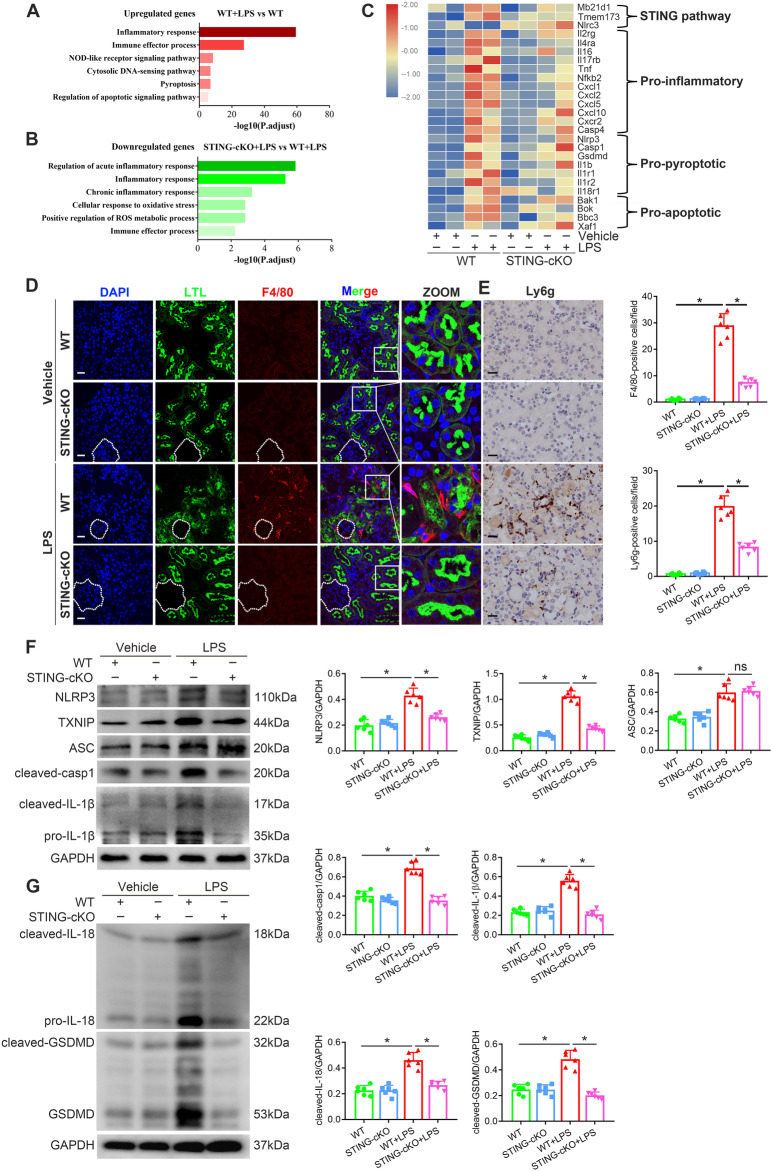
STING-cKO suppressed LPS-induced tubular inflammation and NLRP3 inflammasome activation. **A**, **B** GO analysis showing biologic processes involved by upregulated genes in renal cortices from WT + LPS group vs. WT group (**A**) and downregulated genes in renal cortices from STING-cKO + LPS group vs. WT + LPS group (**B**). **C** Heatmap showing upregulated genes in renal cortices from WT + LPS group (involved in STING pathway, inflammation, pyroptosis and apoptosis) were downregulated by STING-cKO. **D** Representative images and F4/80 (macrophage marker)-positive cells quantitation of immunofluorescence of F4/80, LTL and DAPI in renal tubules from mice per group (original magnification, ×600). Scale bars: 20μm. White outlines indicate glomerulus. **E** Representative images and Ly6g (neutrophil marker)-positive cells quantitation of immunohistochemical staining of Ly6g in renal tubules from mice per group (original magnification, ×800). Scale bars: 20 μm. **F**, **G** Representative western blots and quantitation of NLRP3, TXNIP, ASC, cleaved-casp1, cleaved-IL-1β, cleaved-IL-18, cleaved-GSDMD protein expression in renal cortices from mice per group. **D**–**G** *n* = 6 per group, *P < 0.05 vs. indicated group, ns no significant.

The STING/TBK1/IRF3 pathway plays a key role in innate immune and inflammatory responses. p65, also known as NF-κB, is a key transcription factor of pro-inflammatory genes that could be phosphorylated by TBK1 [25]. As expected, we found that STING, TBK1, IRF3 and p65 were activated in the renal cortices of WT + LPS mice, while STING knockout significantly inhibited these alterations (Fig. S3A). To further investigate the potential mechanisms underlying STING-mediated tubular damage in LPS-induced AKI, an RNA-seq analysis using renal cortices from WT, STING-cKO, WT + LPS and STING-cKO + LPS mice was performed. GO analysis revealed that LPS treatment increased the expression of genes with roles in inflammatory response, immune effector process, NLR signaling pathway, pyroptosis and regulation of apoptotic signaling pathway (Fig. 3A). However, the LPS-activated genes and pathways were suppressed by STING knockout (Fig. 3B). Furthermore, DEGs analysis showed different gene expression patterns among the four groups and listed the key molecules involved in the STING pathway, inflammation, pyroptosis and apoptosis, which were activated by LPS treatment and inhibited by STING knockout (Fig. S3B and Fig. 3C).

Considering that the RNA-seq analysis indicated the closest association between STING and inflammatory response in LPS-induced tubular damage, we assessed it first. The inflammatory cells (macrophages and neutrophils) were identified by F4/80 and Ly6g staining. As shown in (Fig. 3D, E), inflammatory cell infiltration was enhanced in the renal cortices of WT + LPS mice, while it was reduced in STING-cKO + LPS mice. Moreover, the LPS-upregulated mRNA levels of pro-inflammatory cytokines (TNFα, MCP-1, IL-6 and GM-CSF) were markedly reversed by STING knockout (Fig. S3C). Together, the findings demonstrated the vital involvement of STING-mediated inflammation in the tubular damage of LPS-induced AKI.

GO analysis also disclosed that the NLR signals may be an essential link between inflammatory response, immune effector process and oxidative stress (Fig. 3A–C). As mentioned, the NLRP3 inflammasome directly activated by TXNIP (a ROS-activated pro-oxidative molecule) under oxidative stress has gained wide attention in LPS-induced AKI [26]. As displayed in (Fig. 3F, G), STING knockout significantly eliminated the upregulation of NLRP3, TXNIP, cleaved-casp1, cleaved-IL-1β, cleaved-IL-18 and cleaved-GSDMD (except ASC) proteins in WT + LPS mice. IF and IHC assays also disclosed the same tendency of NLRP3 and casp1 protein in renal tubules as western blots (Fig. S3D, E). These results indicated that STING knockout attenuated tubular inflammation and pyroptosis by inhibiting the NLRP3 inflammasome in LPS-induced AKI.

### Effects of STING knockout on LPS-induced tubular STING/mtROS/NLRP3 inflammasome axis activation and apoptosis

**Fig. 4:**
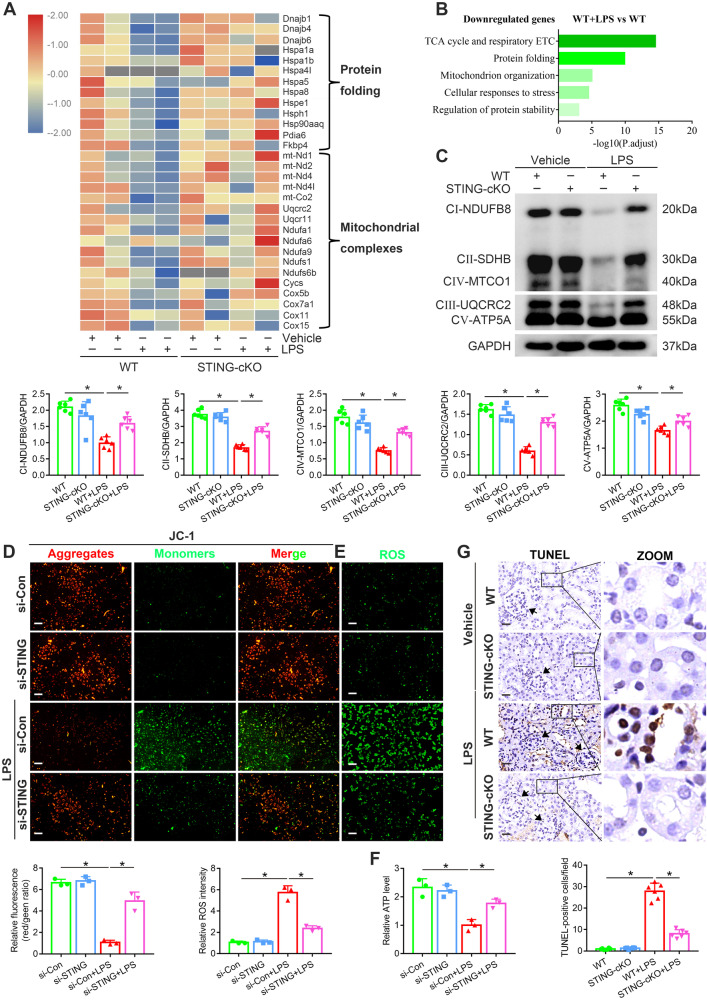
STING-cKO or STING silencing inhibited LPS-induced tubular mtROS overproduction and apoptosis. **A** Heatmap showing the downregulated genes in renal cortices from WT + LPS group (involved in protein folding process and mitochondrial complexes synthesis) were upregulated by STING-cKO. **B** GO analysis showing biologic processes involved by downregulated genes in renal cortices from WT + LPS group vs. WT group. **C** Representative western blots and quantitation of NDUFB8, SDHB, MTCO1, UQCRC2, ATP5A proteins (involved in mitochondrial respiratory chain complexes CI-CIV synthesis) in renal cortices from mice per group, n = 6 per group. **D** Representative images and quantification of MMP (detected by JC-1 fluorescent probe) in HK2 cells per group (original magnification, ×100). Scale bars: 100 μm. **E** Representative images and quantification of ROS generation (marked by DCFH-DA green fluorescent probe) in HK2 cells per group (original magnification, ×100). Scale bars: 100 μm. **F** Representative quantitation of ATP production in HK2 cells per group. **G** Representative images and TUNEL-positive cells quantification of TUNEL staining of renal tubules from mice per group (original magnification, ×800). Scale bars: 20 μm. Black arrows indicate glomerulus, n = 6 per group. **C**–**G** *P < 0.05 vs. indicated group.

Contrary to the above tendency, the key molecules involved in protein folding and mitochondrial complexes synthesis were inhibited by LPS treatment and upregulated after STING knockout (Fig. 4A). LPS treatment in WT mice downregulated the expression of genes with roles in processes such as respiratory electron transport chain (ETC) and protein folding, which were upregulated by STING knockout (Fig. 4B). These data suggested the involvement of oxidative stress and ER stress in the STING-mediated tubular damage of LPS-induced AKI. Mitochondria disorganization and oxidative phosphorylation (OXPHOS) dysfunction both induce mtROS (the vital marker of oxidative stress) overproduction [27]. As shown in (Fig. 4C), the expression of mitochondrial respiratory chain complex proteins composing OXPHOS was inhibited by LPS treatment and recovered after STING knockout. In addition, STING silencing significantly increased MMP (Fig. 4D), ATP (Fig. 4F) and decreased mtROS generation (Fig. 4E) in LPS-treated HK2 cells.

Intriguingly, STING-mediated mtROS overproduction could activate the NLRP3 inflammasome [28]. As shown in (Fig. 3F), the STING knockout inhibited LPS-upregulated TXNIP expression, suggesting that, in addition to the previously reported STING/IRF3-dependent manner, the STING/mtROS/TXNIP axis may be another crucial pathway in NLRP3 inflammasome activation. Moreover, MitoQ (a widely-used mtROS scavenger) dramatically inhibited NLRP3 inflammasome-mediated inflammation and pyroptosis, which were promoted by STING overexpression (Fig. S4A, B). However, the inhibitory effect of MitoQ on the NLRP3 inflammasome, which only reduced TXNIP expression, differed from STING knockout (both reduced NLRP3 and TXNIP expression). These results verified the STING/mtROS/NLRP3 inflammasome axis involved in the tubular inflammation of LPS-induced AKI.

Apoptosis, the hallmark of sepsis, is also linked to the high mortality of septic AKI [29]. Consistent with the data from RNA-seq analysis (Fig. 3A–C), STING knockout significantly alleviated the LPS-induced tubular apoptosis, as evidenced by TUNEL staining (Fig. 4G). mtROS overproduction induced mitochondria-dependent apoptosis by promoting B-cell lymphoma 2 (Bcl2)/Bcl2 associated X (Bax) imbalance, cytochrome c release, and final caspase3 (casp3, the well-known apoptosis marker) recruitment [30]. As shown in (Fig. S5A, B**)**, increased casp3 nuclear translocation, upregulated cleaved-casp3 protein expression and decreased Bcl2/Bax ratio were observed in the WT + LPS group, while STING knockout blocked these pro-apoptotic processes. Meanwhile, the LPS-increased apoptotic level of HK2 cells was reduced by STING silencing (Fig. S5C). These results suggested that STING knockout suppressed tubular apoptosis in LPS-induced AKI.

### Effects of STING knockout on LPS-induced tubular ER stress

**Fig. 5:**
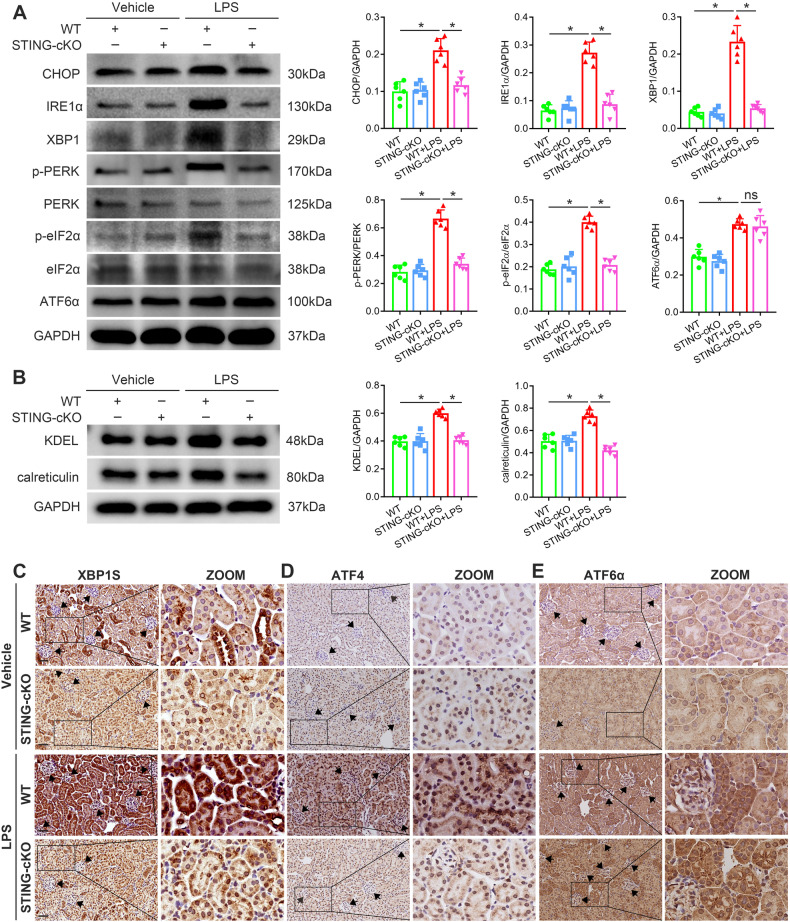
STING-cKO inhibited LPS-induced tubular ER stress. **A**, **B** Representative western blots and quantitation of CHOP, IRE1α, XBP1, p-PERK, p-eIF2α, ATF6α, KDEL, calreticulin protein expression in renal cortices from mice per group (*n* = 6 per group, *P < 0.05 vs. indicated group, ns=no significant). **C**–**E** Representative images of immunohistochemical staining of XBP1S, ATF4 and ATF6α in renal tubules from mice per group (original magnification, ×400). Scale bars: 40 μm. Black arrows indicate glomerulus.

Insufficient protein folding ability activates UPR, which mitigates the ER stress by expanding the ER, increasing the ER folding capacity and transiently shutting down protein translation [31]. More recently, ER stress was characterized as a non-canonical stress/damage-sensing downstream mechanism of STING [32, 33]. In line with the RNA-seq analysis data, LPS treatment activated the two UPR branches (IRE1/X-box binding protein 1 (XBP1)/C/EBP homologous protein (CHOP) and PERK/eukaryotic initiation factor 2α (eIF2α)/ATF4/CHOP pathways), which were blocked by STING knockout (Fig. 5A). ER marker proteins Lys-Asp-Glu-Leu motif (KDEL) and calreticulin also depicted a similar but weaker trend as these two UPR branches (Fig. 5B). IHC assays showed that LPS-increased tubular XBP1S and ATF4 protein levels were reduced by STING knockout (Fig. 5C, D). However, STING knockout had no effects on the LPS-activated ATF6 pathway (Fig. 5A, E), suggesting the potential mechanisms as STING/IRE1 or PERK, but not ATF6 axis for tubular damage in LPS-induced AKI. Additionally, LPS treatment in HK2 cells promoted cytoplasmic translocation of XBP1S, ATF4 and ATF6α, which were recovered by STING silence (excepted ATF6α), further supporting in-vivo results (Fig. S6A–C).

### STING contributed to LPS-induced inflammation, pyroptosis and mtROS overproduction in an NLRP3-independent manner by activating ER stress

**Fig. 6:**
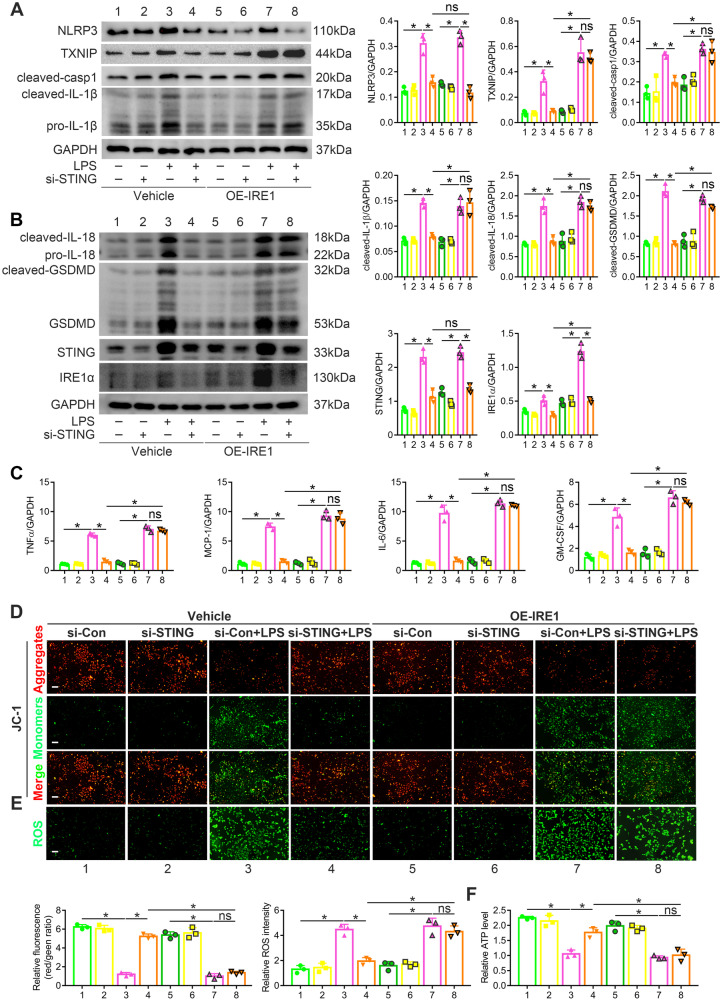
IRE1 overexpression activated inflammation, pyroptosis and mtROS overproduction in LPS-treated HK2 cells under STING silencing. **A**, **B** Representative western blots and quantitation of NLRP3, TXNIP, cleaved-casp1, cleaved-IL-1β, cleaved-IL-18, cleaved-GSDMD, STING, IRE1α protein expression in HK2 cells per group. **C** Representative quantitation of TNFα, MCP-1, IL-6, GM-CSF mRNA levels (fold change) in HK2 cells per group. **D** Representative images and quantification of MMP (detected by JC-1 fluorescent probe) in HK2 cells per group (original magnification, ×100). Scale bars: 100μm. **E** Representative images and quantification of ROS generation (marked by DCFH-DA green fluorescent probe) in HK2 cells per group (original magnification, ×100). Scale bars: 100 μm. **F** Representative quantitation of ATP production in HK2 cells per group. **A**–**F** *P < 0.05 vs. indicated group, ns no significant.

Interestingly, recent studies have highlighted the disordered ER stress/mitochondrial dysfunction/NLRP3 inflammasome axis as the pathological basis of various inflammatory diseases. Thus, we studied whether NLRP3 inflammasome activation by ER stress depends on STING in LPS-induced AKI. We treated HK2 cells with LPS after transfecting si-STING and OE-IRE1 to observe the STING/ER stress/NLRP3 axis alterations. As shown in (Fig. 6A, B), IRE1 overexpression significantly promoted LPS-activated NLRP3 inflammasome-mediated inflammation and pyroptosis, as evidenced by the increased TXNIP, cleaved-casp1, cleaved-IL-1β, cleaved-IL-18 and cleaved-GSDMD protein expression, even though STING was silenced. However, STING silence counteracted the upregulation of STING, NLRP3 and IRE1α protein triggered by LPS treatment, even though IRE1 was overexpressed (Fig. 6A, B), suggesting that NLRP3 and IRE1 lie downstream of STING, and NLRP3 may be activated in the previously reported STING/IRF3-dependent manner but not ER stress. Similarly, LPS-enhanced TNFα, MCP-1, IL-6 and GM-CSF transcription was inhibited by STING silencing and upregulated after IRE1 overexpression (Fig. 6C). Furthermore, compared to the si-STING + LPS group, the si-STING + OE-IRE1 + LPS group showed decreased MMP, reduced ATP production and increased mtROS generation (Fig. 6D–F). These data suggested that the LPS-induced STING/ER stress/mtROS axis promoted tubular NLRP3 inflammasome-mediated inflammation and pyroptosis without activating NLRP3, possibly cause by TXNIP increase.

To verify the regulatory effect of STING-mediated ER stress on NLRP3 inflammasome, we also transfected si-STING and OE-NLRP3 in HK2 cells before LPS treatment. Compared with the LPS group, the si-STING + LPS and OE-NLRP3 + si-STING + LPS groups showed decreased phosphorylation levels of TBK1, IRF3 and p65 proteins (Fig. S7A). Additionally, NLRP3 overexpression could not activate IRE1α, XBP1, PERK and eIF2α after STING silencing, even with LPS treatment (Fig. S7B). These data suggested that STING mediated LPS-induced tubular ER stress in an NLRP3 inflammasome-independent manner, which further proved the STING/ER stress/mtROS/NLRP3 inflammasome axis in LPS-induced AKI.

## Discussion

In the past century, most studies in the field of septic AKI focused on bacterial invasion-related hemodynamic factors. Until recently, the involvement of dysregulated innate immune and inflammatory responses in septic AKI was disclosed [34, 35]. In fact, tubular cells constitutively express PRRs and tend to be the source of inflammatory cytokines, which recruit immune cells in LPS-induced AKI (mainly neutrophils and macrophages), in turn secrete inflammatory cytokines triggering further tubular damage to form a deterioration loop, rather than the main victim of intrarenal inflammation [36–38]. Thus, exploring the key molecular mechanisms of tubular cell-derived innate immune and inflammatory responses may offer a breakthrough for septic AKI. STING, also known as human transmembrane protein 173 (TMEM173), has been demonstrated repeatedly to implicate the inflammatory damage of various diseases (e.g., nonalcoholic steatohepatitis, Parkinson’s disease, aortic aneurysm and dissection) [39–41]. The unique location (MAMs) not only makes STING a detecting station of cytosolic invaders, but also allows it to respond to and amplify alarm signals from the ER and mitochondria [7, 42, 43].

In our study, tubular cells induced by LPS exhibited significant STING/TBK1/IRF3 pathway activation, which may correlate with renal dysfunction and histological tubular damage. The above LPS-induced effects were dramatically relieved by STING-cKO. Thus, STING could be a novel critical molecule involved in the progression of LPS-induced AKI, but its precise role requires further exploration. Increasing reports have shown the interplay between the STING/TBK1/IRF3 or p65 pathway and the NLRP3 inflammasome [19, 44]. In our study, RNA-seq analysis data suggested that STING is closely related to pro-inflammatory, pro-pyroptotic, pro-apoptotic, protein folding and mitochondria complex synthesis pathways. Moreover, NLR signaling is the pathway most strongly associated with STING to mediate inflammatory response, immune effector process and oxidative stress. The NLR family proteins were also identified as cytoplasmic PRRs [45]. Recently, NLRP3 inflammasome activation from non-immune cells was a potent trigger to immune cells infiltration [46]. The concepts of neutrophil extracellular traps and macrophage M1 polarization were proposed that regulated by NLRP3 inflammasome to co-mediate inflammatory damage [47, 48]. NLRP3 physiologically localizes on the ER, while activated NLRP3 and its adapter ASC strategically redistribute to MAMs to sense alarm signals emanating from mitochondria, which initiates inflammatory cytokine cascade and pyroptosis [49]. A recent study unveiled that STING activated NLRP3 in an IRF3-dependent manner [50]. Beyond ASC, the newfound NLRP3-binding partner TXNIP also relocates to mitochondria/MAMs in response to oxidative stress (particularly mtROS) [51]. mtROS could promote the dissociation of TXNIP with TXNIP-thioredoxin, and trigger the association of TXNIP with NLRP3 [52]. In our study, reducing mtROS suppressed the STING-activated NLRP3 inflammasome through inhibited TXNIP expression, which differed from STING-cKO (which inhibited both NLRP3 and TXNIP expression). We thus hypothesized that mtROS could promote NLRP3 inflammasome activation by activating TXNIP in LPS-induced AKI, even with STING knockout.

During oxidative stress, mtROS are generated from the mitochondrial respiratory chain where the electrons cannot be efficiently coupled with mitochondrial complexes I and III [53, 54]. The disproportionate mtROS product from mitochondria is a driving force for apoptosis [55, 56]. In our study, STING-cKO dramatically alleviated LPS-induced tubular oxidative stress. STING-cKO or STING silencing suppressed LPS-induced tubular cell apoptosis. As known, ER stress and oxidative stress are both derived from MAMs. The signals are released from the ER, across MAMs, and uptaken by mitochondria, leading to pro-survival or pro-apoptotic adaptations [57, 58]. mtROS are also generated downstream of and as a consequence of ER stress. In mouse embryonic fibroblasts (MEFs), TXNIP protein induction was completely abolished with IRE1 or PERK ablation, but unaffected with ATF6 ablation [59]. Intriguingly, the three ER-resident UPR sensors IRE1, PERK and ATF6 were verified to increase MAMs formation during ER stress [60, 61]. The common location (MAMs) may provide a platform for STING engagement to trigger ER stress, oxidative stress and the NLRP3 inflammasome. In our study, STING-cKO significantly inhibited LPS-induced tubular ER stress in IRE1 and PERK pathways but not in the ATF6 pathway. Moreover, despite STING silencing, IRE1 overexpression increased mtROS generation and upregulated TXNIP to activate the NLRP3 inflammasome. However, NLRP3 overexpression did not affect the STING/TBK1/IRF3 pathway and ER stress (IRE1 and PERK pathways).

The crosstalk of ER stress, mtROS, and NLRP3 inflammasome between STING in septic AKI were summarized. Otherwise, their connection with apoptosis was still unclear. Multiple studies partially confirmed the close connection between STING/ER stress/mtROS/NLRP3 inflammasome axis and apoptosis: The caspase8/3-induced apoptosis was regarded as an intrinsic part of NLRP3 inflammasome, which could be activated by enhanced mtROS production [62, 63]; Various ROS-promoting enzymes that participate in the UPR process were identified, mtROS induction regulated by UPR and in turn aggravate UPR, which exacerbate each other and then promote apoptosis [64–66]; Moreover, the activation of STING/ER stress, STING/NLRP3, and STING/mtROS signaling all verified to prime apoptosis [32, 67, 68].

Taken together, this study highlights the critical role of the STING/ER stress/mtROS/NLRP3 inflammasome axis in the tubular damage of LPS-induced AKI. As shown in the graphical abstract, in healthy tubular cells, STING exists in the MAMs. In tubular cells exposed to LPS, STING translocates from MAMs. On the one hand, STING promotes IRF3 phosphorylation to upregulate NLRP3 expression. On the other hand, STING triggers the IRE1/XBP1 pathway of ER stress to induce mtROS overproduction, which enhances the association of TXNIP with NLRP3. Eventually, the NLRP3 inflammasome is assembled to initiate the maturation and secretion of IL-1β, IL-18 and GSDMD, resulting in tubular inflammation, pyroptosis and apoptosis.

Notably, in our study, STING-cKO showed no significant effects on LPS-activated ATF6 expression. Regarding the activation mechanism, ATF6 is cleaved to the active form, while IRE1 and PERK switch from monomers to oligomers that allow autophosphorylation and activation [69, 70]. The unique activation patterns of ATF6 may explain its absence from STING-related ER stress. Another interesting finding in our study was that LPS treatment also promoted the transcription of the TNFα, MCP-1, IL-6 and GM-CSF genes, which are not direct effectors of the NLRP3 inflammasome. We speculated that IL-1β upregulated these inflammatory cytokines, further amplifying tubular inflammation in an NLRP3-dependent manner. IL-1β exerts multiple effects on extensive inflammatory events. Thus, blocking IL-1 receptors could dampen most signs of tissue inflammation [71, 72]. Lastly, PANoptosis, an emerging concept highlighting the crosstalk and coordination between pyroptosis, apoptosis and necroptosis, may facilitate our insight into innate immune and inflammatory cell death [73]. More recently, STING-dependent PANoptosis was reported in acute lung inflammation/ARDS, suggesting the central regulatory effects of STING on cell fate [74]. However, whether the STING/ER stress/mtROS/NLRP3 inflammasome axis is an essential pathway leading to PANoptosis remains to be further explored.

## Conclusion

In conclusion, this is the first study to disclose that STING contributes to tubular inflammation and pyroptosis by activating ER stress in LPS-induced AKI. STING knockout attenuates LPS-induced tubular cell damage by suppressing the STING/ER stress/mtROS/NLRP3 inflammasome axis. Thus, our study provides essential evidence suggesting that targeting the STING pathway serve as a novel therapeutic strategy for septic AKI.

### Supplementary information

Supplementary Information

Original Data File

## Data Availability

All data needed to evaluate the conclusions are present in the paper and the supplementary information. Additional data supporting the findings in this study are available from the corresponding author upon reasonable request.
