# Supplementary material for: STING contributes to lipopolysaccharide-induced tubular cell inflammation and pyroptosis by activating endoplasmic reticulum stress in acute kidney injury
Source: Cell Death Dis. 2024 Mar 14;15(3):217. doi: 10.1038/s41419-024-06600-1 (PMC10940292; doi:10.1038/s41419-024-06600-1)
Supplement: Supplementary file 1 — Supplementary Information [file 41419_2024_6600_MOESM1_ESM.docx]

**Supporting information**

**Table S1. Information for the primary antibodies used in western blot analysis.**

| **Antibody** | **Company** | **Catalog** | **Application** |
| --- | --- | --- | --- |
| GAPDH | Santa Cruz (United States) | sc-32233 | 1:10000 |
| IRF3 | Santa Cruz (United States) | sc-33641 | 1:200 |
| CHOP | Santa Cruz (United States) | sc-7351 | 1:200 |
| PERK | Santa Cruz (United States) | sc-377400 | 1:100 |
| KDEL | Santa Cruz (United States) | sc-58774 | 1:200 |
| STING | CST (United States) | D2P2F | 1:1000 |
| p-TBK1 | CST (United States) | #5483 | 1:1000 |
| p-IRF3 | CST (United States) | #29047 | 1:1000 |
| p-p65 | CST (United States) | #93H1 | 1:1000 |
| p65 | CST (United States) | #8242S | 1:1000 |
| IRE1α | CST (United States) | #3294 | 1:1000 |
| p-PERK | CST (United States) | #3179 | 1:1000 |
| p-eIF2α | CST (United States) | #3398 | 1:1000 |
| eIF2α | CST (United States) | #9722 | 1:1000 |
| NLRP3 | CST (United States) | D4D8T | 1:1000 |
| TBK1 | Abcam (United Kingdom) | ab40676 | 1:1000 |
| XBP1 | Abcam (United Kingdom) | ab37152 | 1:1000 |
| Total OXPHOS rodent WB antibody cocktail | Abcam (United Kingdom) | ab110413 | 1:1000 |
| Bcl2 | Abcam (United Kingdom) | ab32124 | 1:1000 |
| cleaved-casp3 | Abcam (United Kingdom) | ab32042 | 1:500 |
| calreticulin | Abcam (United Kingdom) | ab92516 | 1:1000 |
| TXNIP | ABclonal (China) | A9342 | 1:1000 |
| ASC | ABclonal (China) | A1170 | 1:500 |
| cleaved-casp1 | ABclonal (China) | A23429 | 1:1000 |
| pro-/cleaved-IL-1β | ABclonal (China) | A16288 | 1:500 |
| pro-/cleaved-IL-18 | ABclonal (China) | A20473 | 1:500 |
| GSDMD/cleaved-GSDMD | ABclonal (China) | A24476 | 1:1000 |
| ATF6α | Proteintech (China) | 24169-1-AP | 1:2000 |
| Bax | Proteintech (China) | 60267-1-lg | 1:5000 |

**Table S2. Information for the primary antibodies used in IHC.**

| **Antibody** | | **Company** | | **Catalog** | | **Application** | |
| --- | --- | --- | --- | --- | --- | --- | --- |
| STING | | CST (United States) | | D2P2F | 1:100 |  |  |
| Ly6g | | Abcam (United Kingdom) | | ab238132 | 1:2000 |  |  |
| ATF4 | | Abcam (United Kingdom) | | ab31390 | 1:50 |  |  |
| cleaved-casp1 | | Proteintech (China) | | 22915-1-AP | 1:200 |  |  |
| XBP1S | | Proteintech (China) | | 24868-1-AP | 1:100 |  |  |
| ATF6α | | Proteintech (China) | | 24169-1-AP | 1:50 |  |  |

**Table S3. Information for the primary antibodies used in IF assay.**

| **Antibody** | **Company** | **Catalog** | **Application** |
| --- | --- | --- | --- |
| STING | CST (United States) | D2P2F | 1:100 |
| F4/80 | CST (United States) | #70076 | 1:250 |
| cleaved-casp3 | CST (United States) | #9664 | 1:500 |
| NLRP3 | ImmunoWay (United States) | YT5382 | 1:100 |
| KDEL | Santa Cruz (United States) | sc-58774 | 1:50 |
| ATF4 | Abcam (United Kingdom) | ab31390 | 1:100 |
| XBP1S | Proteintech (China) | 24868-1-AP | 1:100 |
| ATF6α | Proteintech (China) | 24169-1-AP | 1:50 |

**Table S4. Primers for real-time quantitative PCR**

| **Gene** | **Species** | **Forward primer** | **Reverse primer** |
| --- | --- | --- | --- |
| TNFα | Mouse | ACTGAACTTCGGGGTGATCGGT | TGGTTTGCTACGACGTGGGCTA |
| MCP-1 | Mouse | TGGCTCAGCCAGATGCAGT | CCAGCCTACTCATTGGGATCA |
| IL-6 | Mouse | TCCAGTTGCCTTCTTGGGAC | GTGTAATTAAGCCTCCGACTTG |
| GM-CSF | Mouse | CCAGCTCTGAATCCAGCTTCTC | TCTCTCGTTTGTCTTCCGCTGT |
| GAPDH | Mouse | ACTCCACTCACGGCAAATTC | TCTCCATGGTGGTGAAGACA |
| TNFα | Human | CCTCTCTCTAATCAGCCCTCTG | GAGGACCTGGGAGTAGATGAG |
| MCP-1 | Human | CAGCCAGATGCAATCAATGCC | TGGAATCCTGAACCCACTTCT |
| IL-6 | Human | GGTACATCCTCGACGGCATCT | GTGCCTCTTTGCTGCTTTCAC |
| GM-CSF | Human | GCCAGCCACTACAAGCAGCAC | CAAAGGGGATGACAAGCAGAAAG |
| GAPDH | Human | CCTCAACGACCACTTTGTCA | TTACTCCTTGGAGGCCATGT |


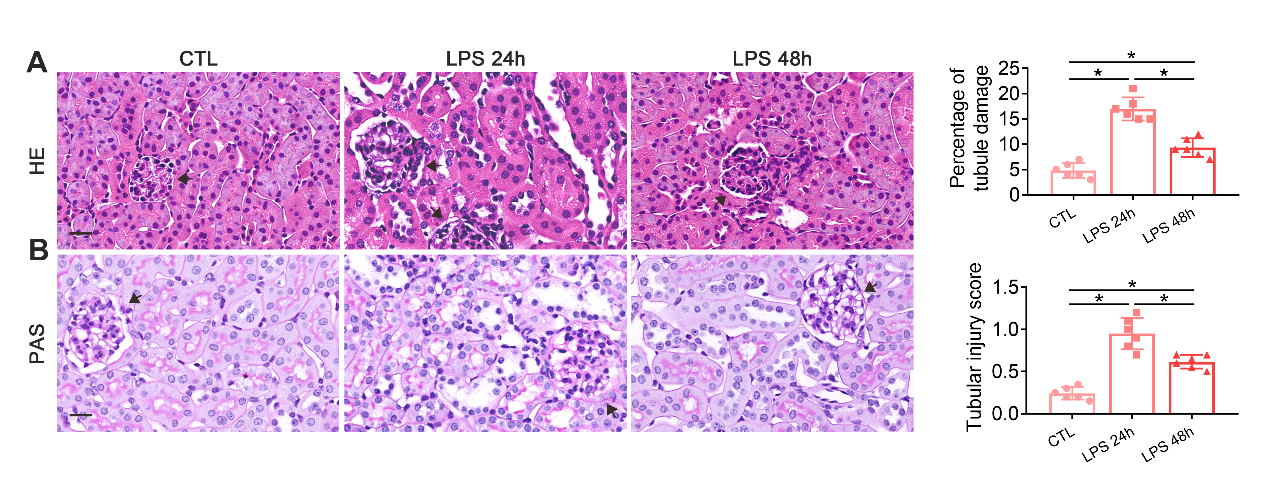


**Figure S1. Renal pathological changes of mice treated with LPS 24 or 48 h.** (A, B) Representative images of HE and PAS staining of renal tubules from mice i.p.-injected with LPS (10 mg/kg) for 0, 24, 48h. (original magnification, ×800). Scale bars: 20μm. Black arrows indicate glomerulus. n=6 per group, *P＜0.05 vs. indicated group.


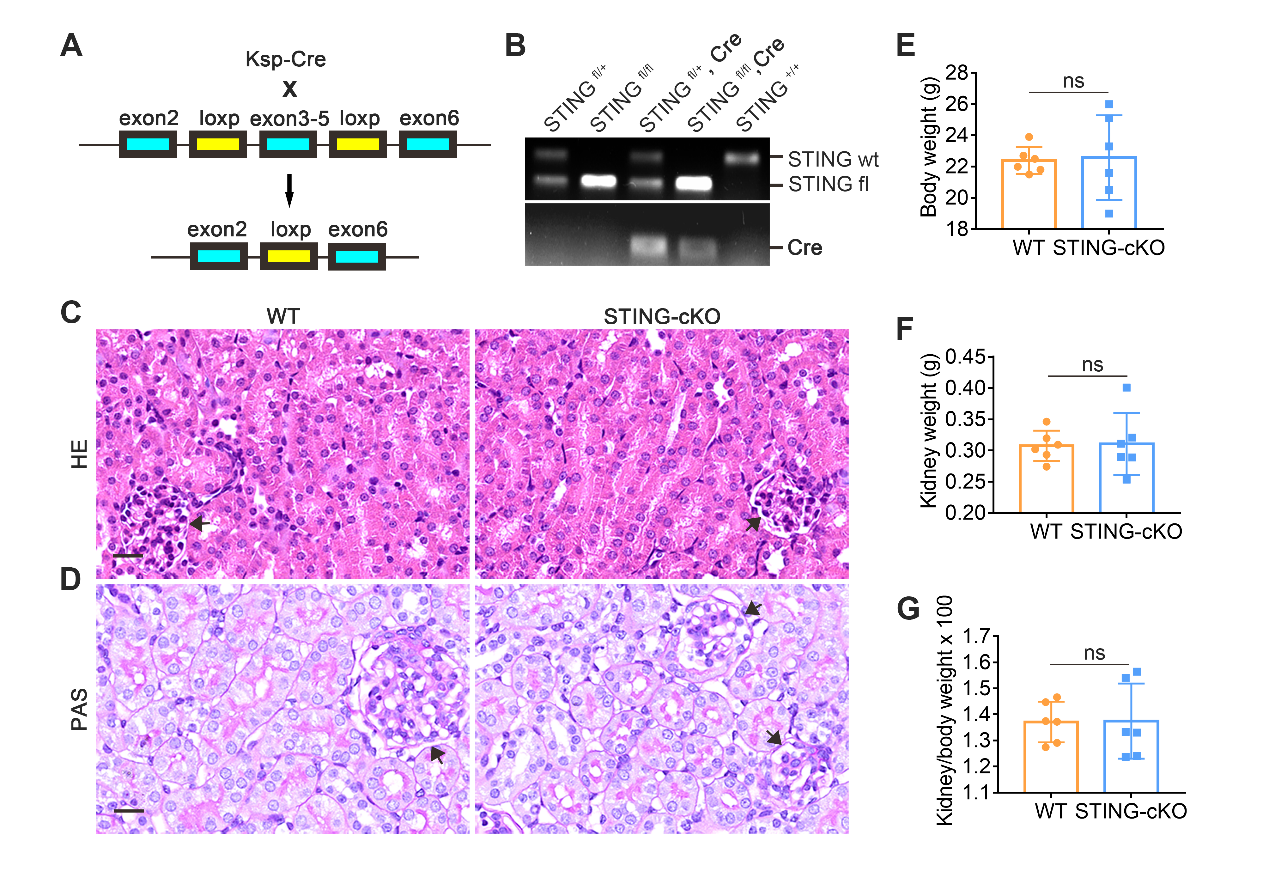


**Figure S2. Generation strategy and pathophysiologic changes of STING-cKO mice.** (A) Strategy for generating STING-cKO mice. (B) Genotyping the mice by PCR analysis of genomic DNA, from left to right, are STING^fl/+^; STING^fl/fl^; STING^fl/+^, Cre; STING^fl/fl^, Cre and STING^+/+^ genotype. (C, D) Representative images of HE and PAS staining of renal tubules from WT mice and STING-cKO mice (original magnification, ×800). Scale bars: 20μm. Black arrows indicate glomerulus. (E–G) Representative quantitation of body weight, kidney weight and the ratio of kidney weight/body weight of WT mice and STING-cKO mice, n=6 per group, ns=not significant.


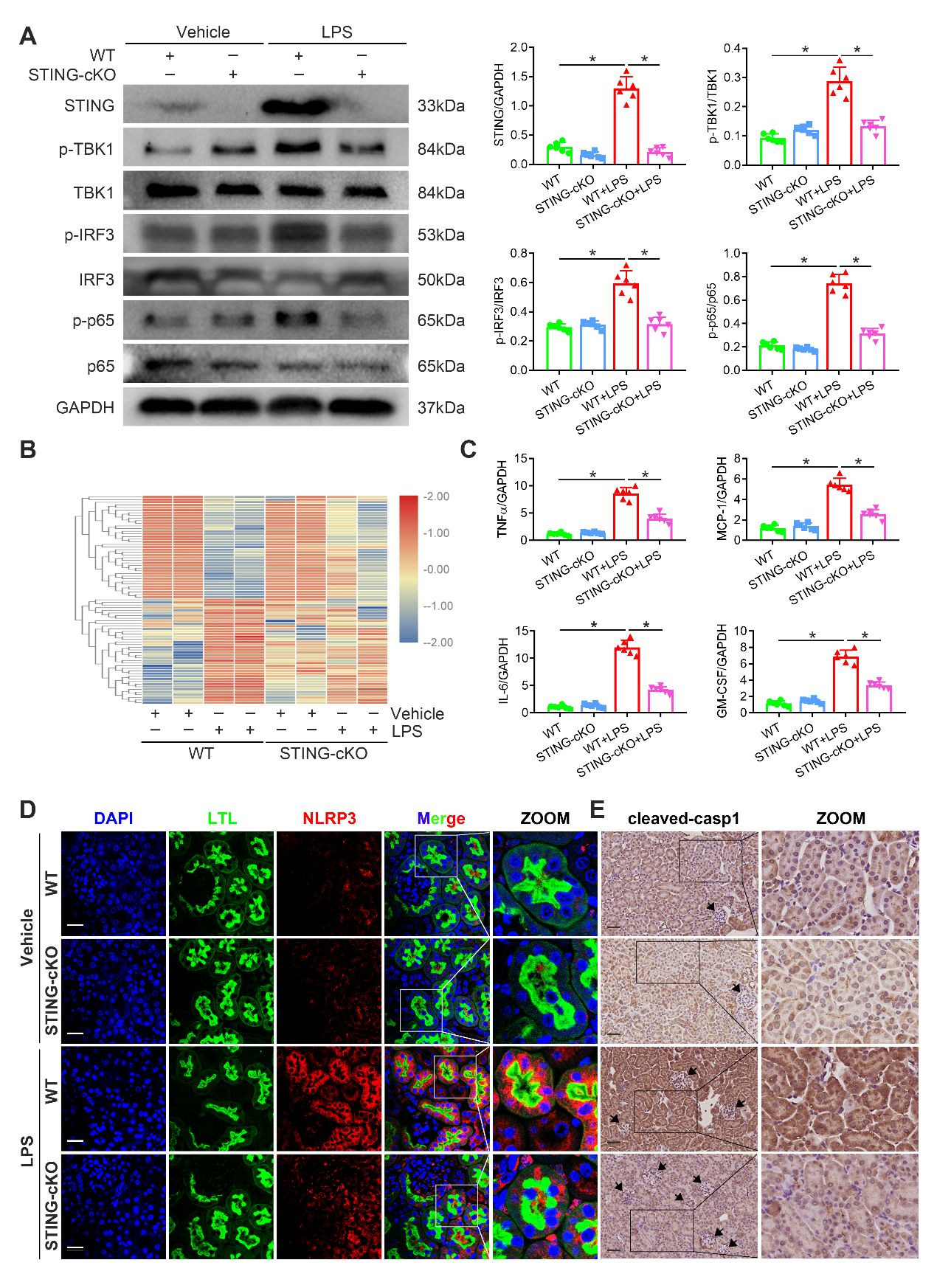


**Figure S3. STING-cKO inhibited the LPS-activated tubular STING/TBK1/IRF3 or p65 pathway and NLRP3 inflammasome activation.** (A) Representative western blots and quantitation of STING, p-TBK1, p-IRF3, p-p65 protein expression in renal cortices from mice per group. (B) Heatmap showing different gene expression patterns in renal cortices from mice per group. (C) Representative quantitation of TNFα, MCP-1, IL-6, GM-CSF mRNA levels (fold change) in renal cortices from mice per group. (A, C) n=6 per group, *P＜0.05 vs. indicated group. (D) Representative images of NLRP3, LTL and DAPI immunofluorescence in renal tubules from mice per group (original magnification, ×1000). Scale bars: 20μm. (E) Representative images of cleaved-casp1 immunohistochemical staining in renal tubules from mice per group (original magnification, ×400). Scale bars: 40μm. Black arrows indicate glomerulus.


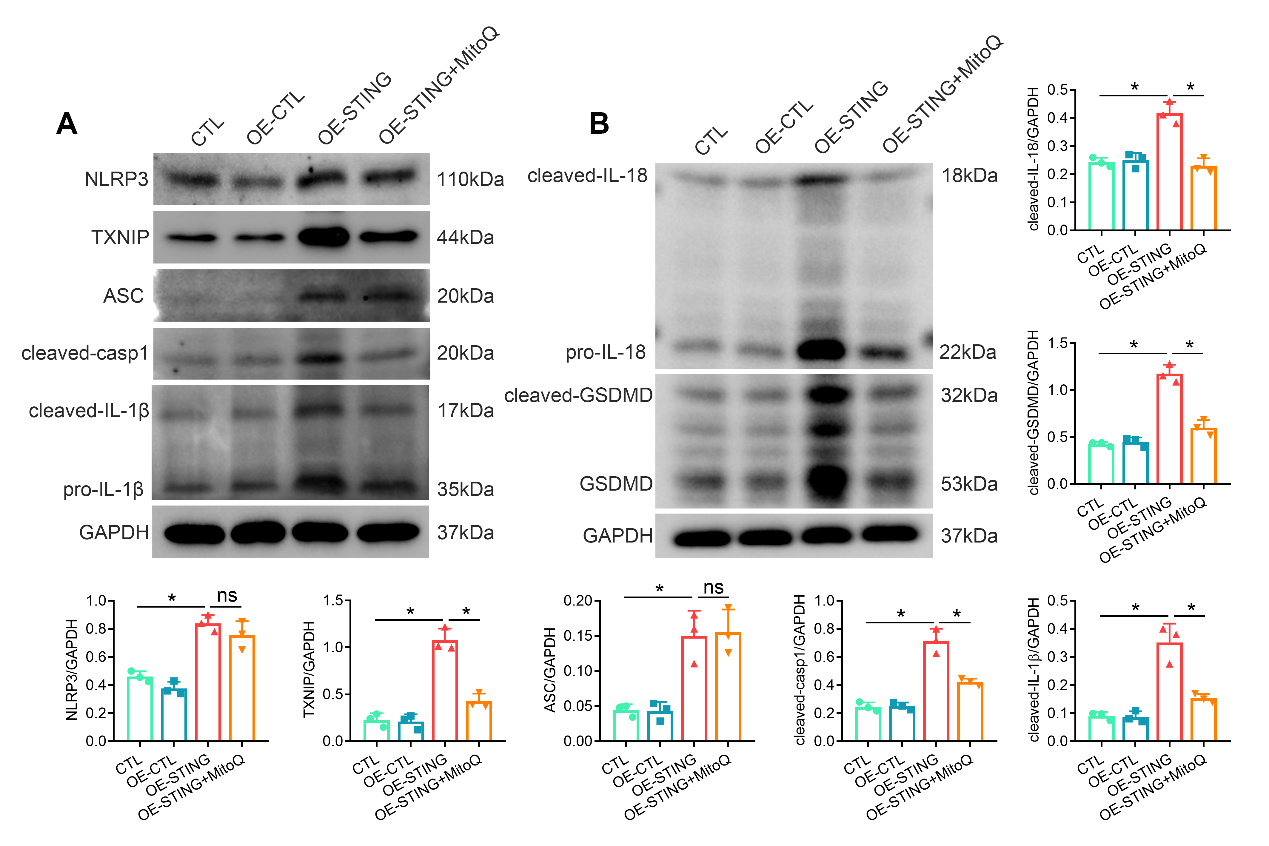


**Figure S4. MitoQ treatment suppressed the STING-activated NLRP3 inflammasome-mediated inflammation and pyroptosis.** (A, B) Representative western blots and quantitation of NLRP3, TXNIP, ASC, cleaved-casp1, cleaved-IL-1β, cleaved-IL-18, cleaved-GSDMD proteins in HK2 cells per group (*P＜0.05 vs. indicated group, ns=not significant).


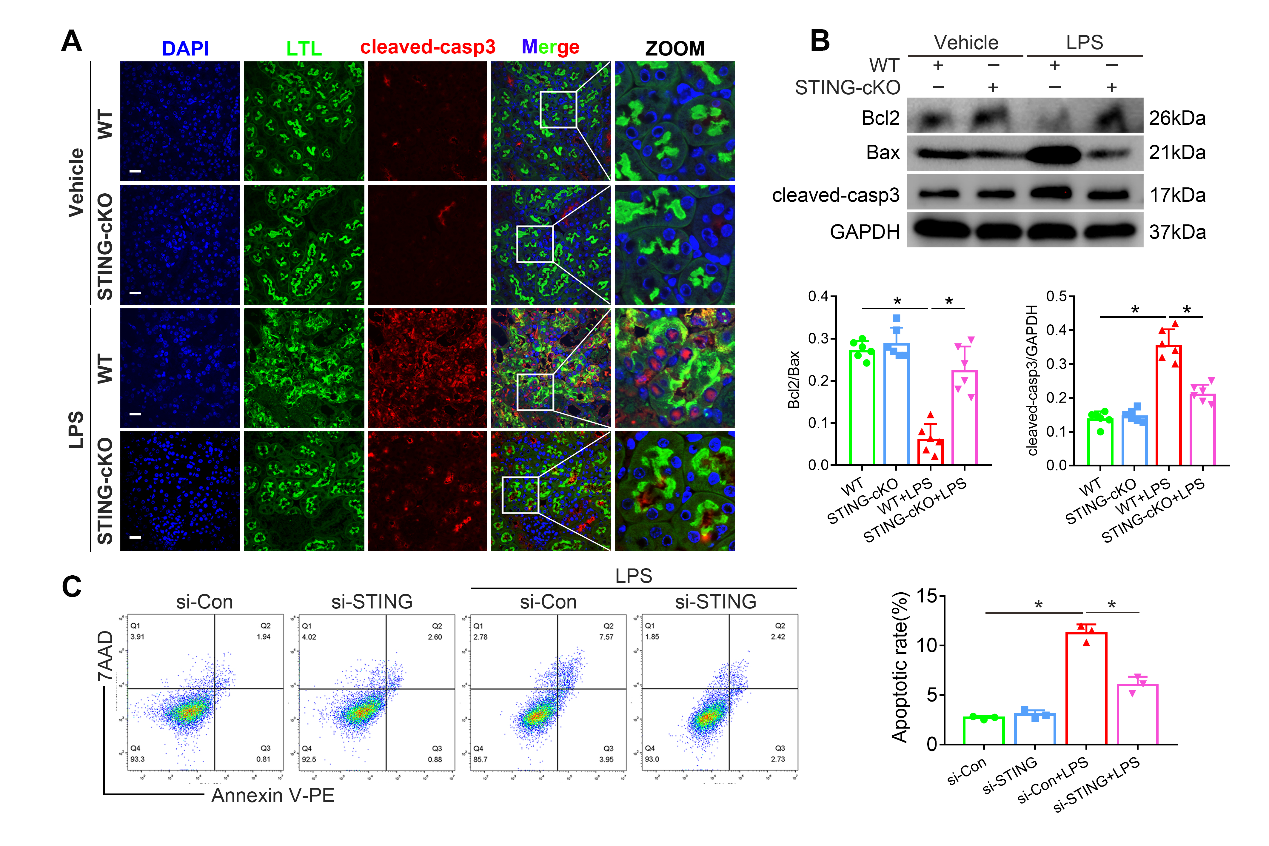


**Figure S5. STING-cKO or STING silence suppressed LPS-induced apoptosis of tubular cells.** (A) Representative immunofluorescence images of cleaved-casp3, LTL and DAPI in renal tubules from mice per group (original magnification, ×600). Scale bars: 20μm. (B) Representative western blots and quantitation of Bcl2, Bax, cleaved-casp3 expression in renal cortices from mice per group (n=6 per group). (C) Flow cytometry analysis of apoptosis in HK2 cells and quantitation per group. (B, C) (*P＜0.05 vs. indicated group).


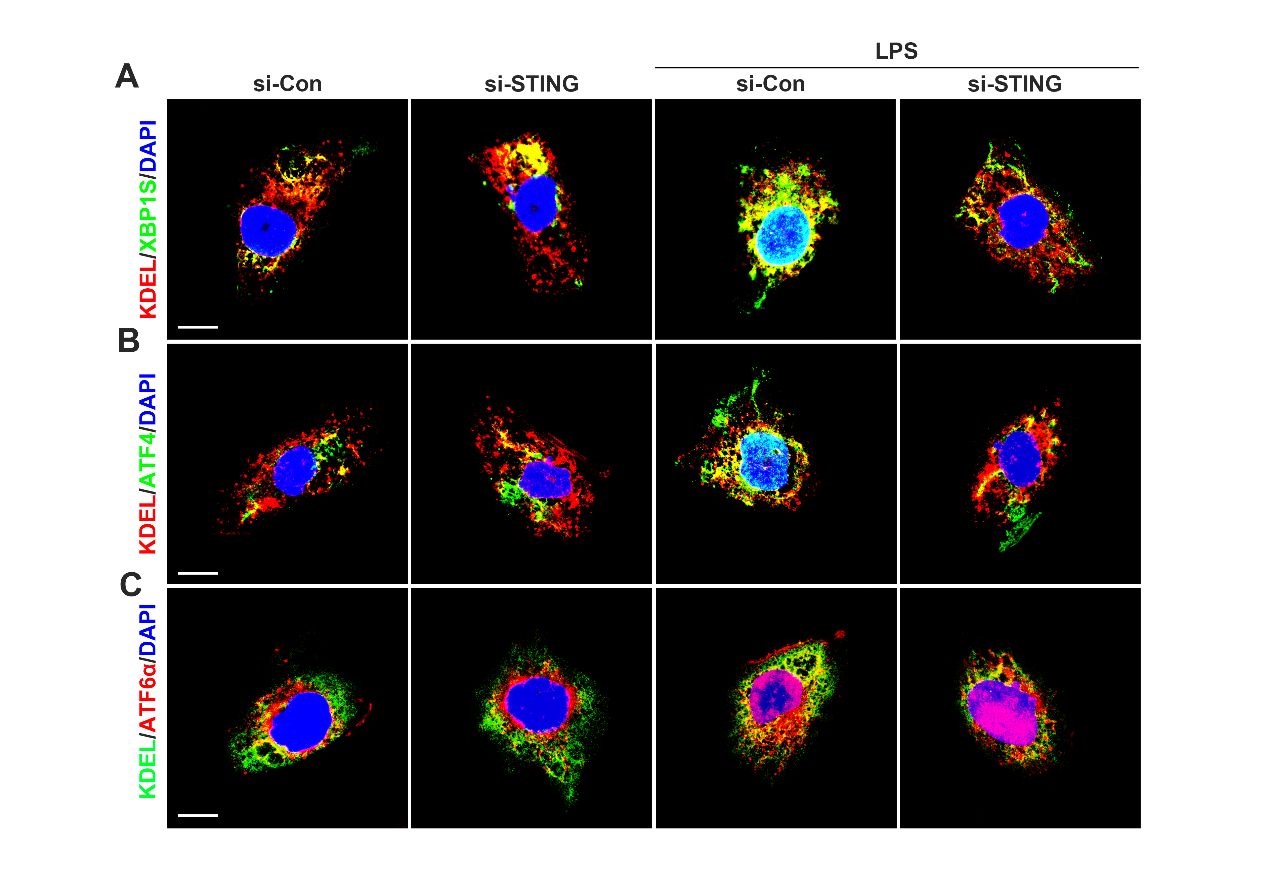


**Figure S6. STING silence inhibited LPS-induced ER stress of HK2 cells.** (A-C) Representative immunofluorescence images of KDEL, XBP1S/ATF4/ATF6α and DAPI in HK2 cells per group (original magnification, ×1000). Scale bars: 20μm.


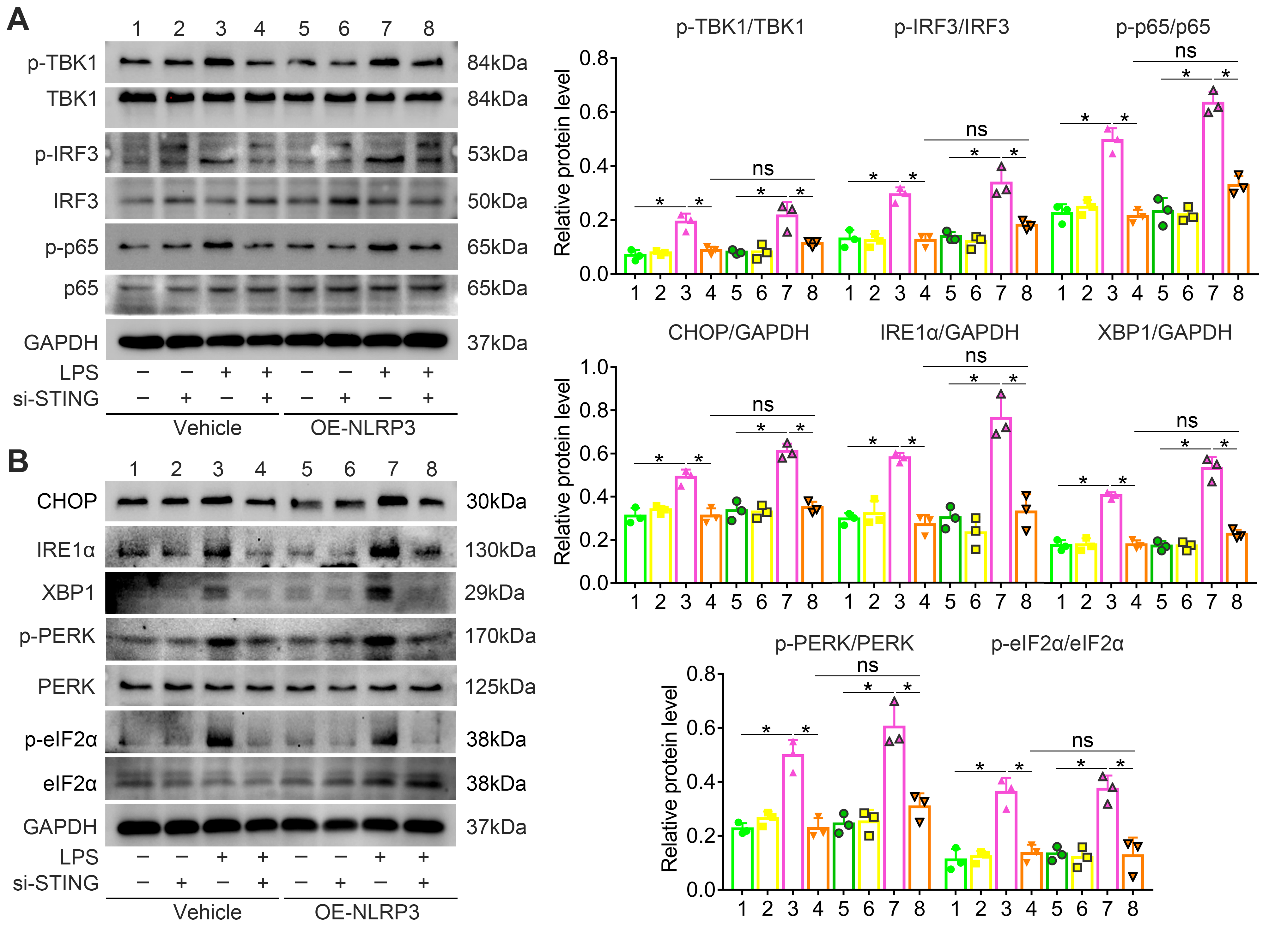


**Figure S7. NLRP3 overexpression had no effects on the STING/TBK1/IRF3 or p65 pathway and ER stress in LPS-treated HK2 cells under STING silencing.** (A) Representative western blots and quantitation of p-TBK1, p-IRF3, p-p65 protein expression in HK2 cells per group. (B) Representative western blots and quantitation of CHOP, IRE1α, XBP1, p-PERK, p-eIF2α protein expression in HK2 cells per group. (A, B) *P＜0.05 vs. indicated group, ns=no significant.
