# Supplementary material for: STING contributes to lipopolysaccharide-induced tubular cell inflammation and pyroptosis by activating endoplasmic reticulum stress in acute kidney injury
Source: Cell Death Dis. 2024 Mar 14;15(3):217. doi: 10.1038/s41419-024-06600-1 (PMC10940292; doi:10.1038/s41419-024-06600-1)

**Original western blots 1**


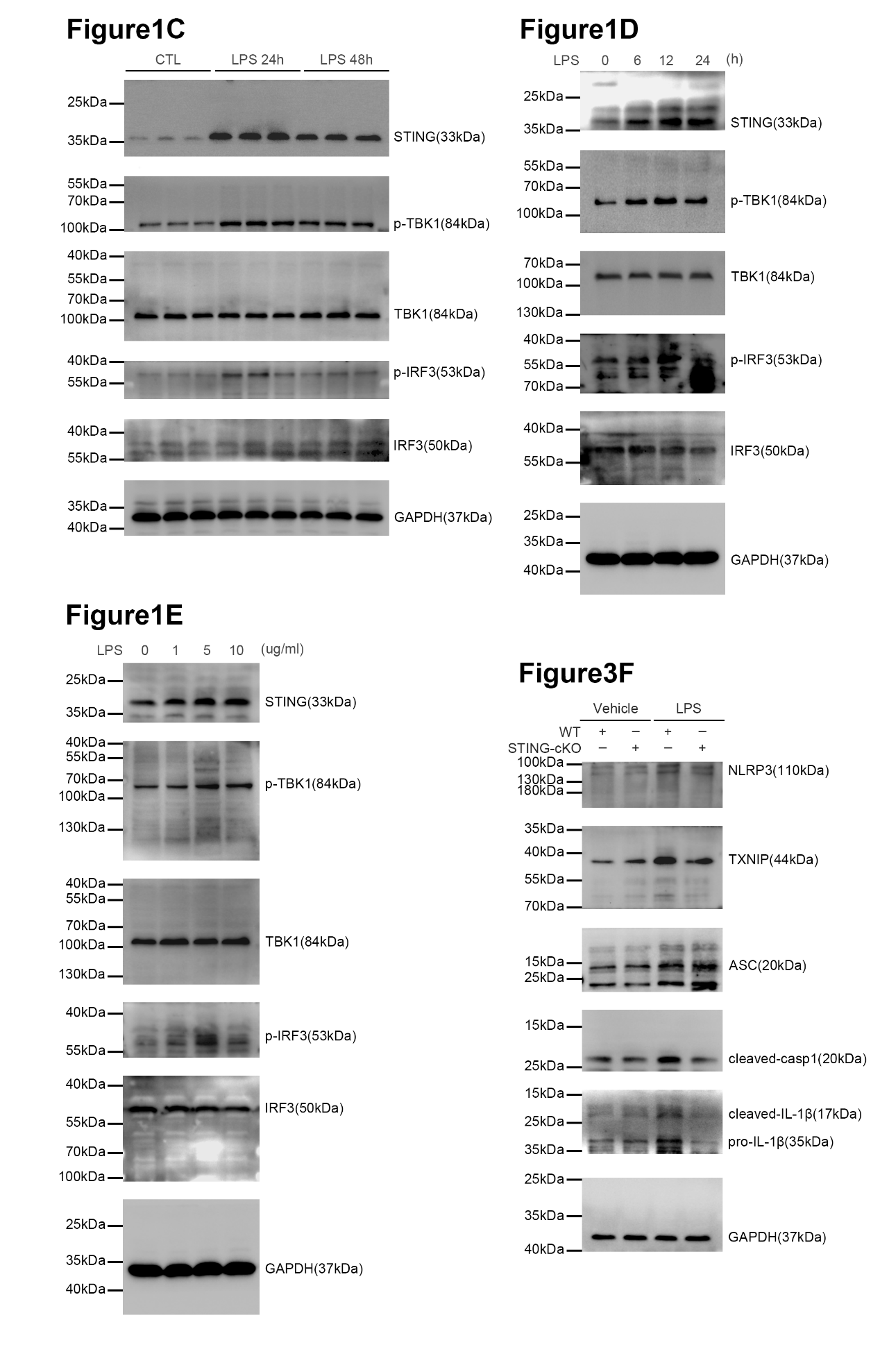


**Original western blots 2**


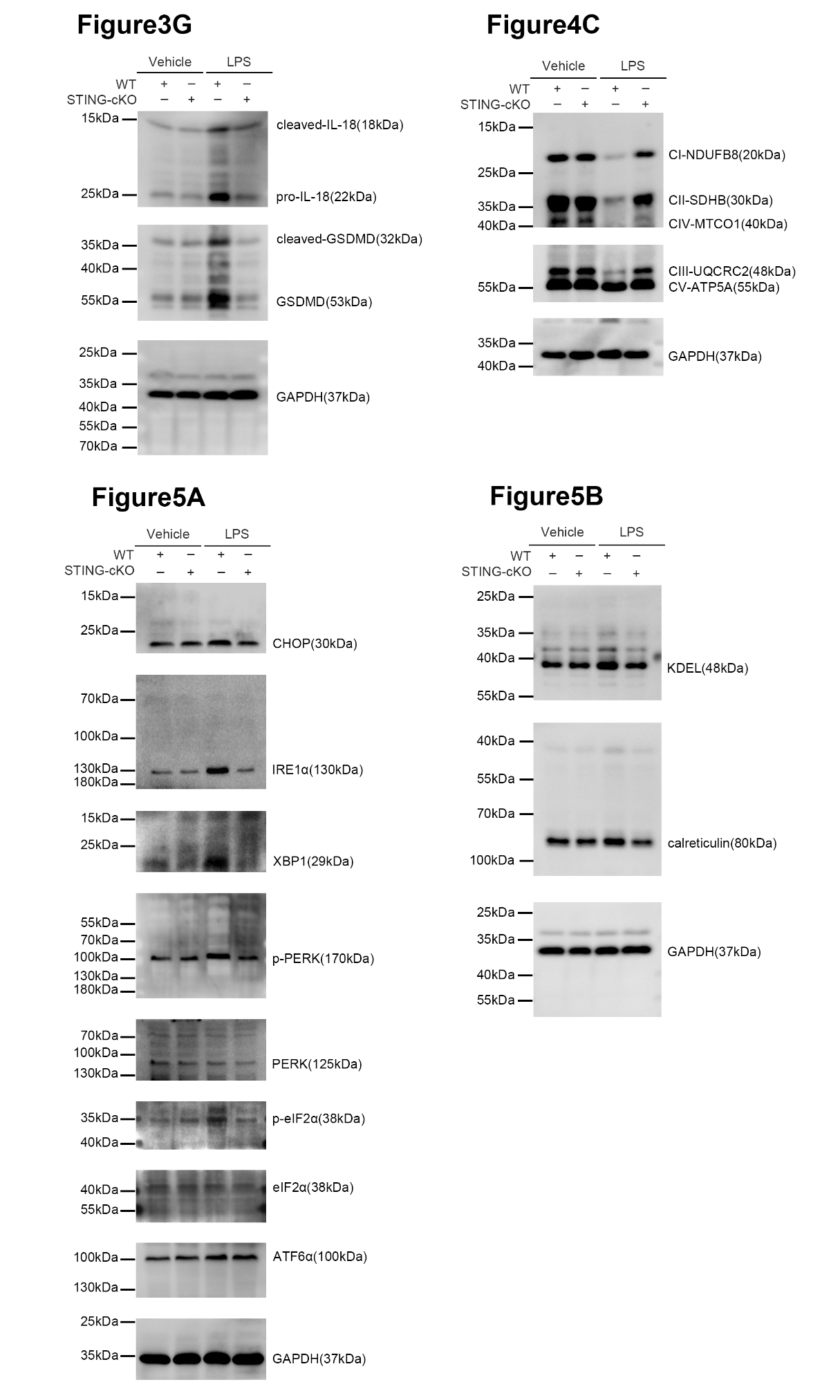


**Original western blots 3**


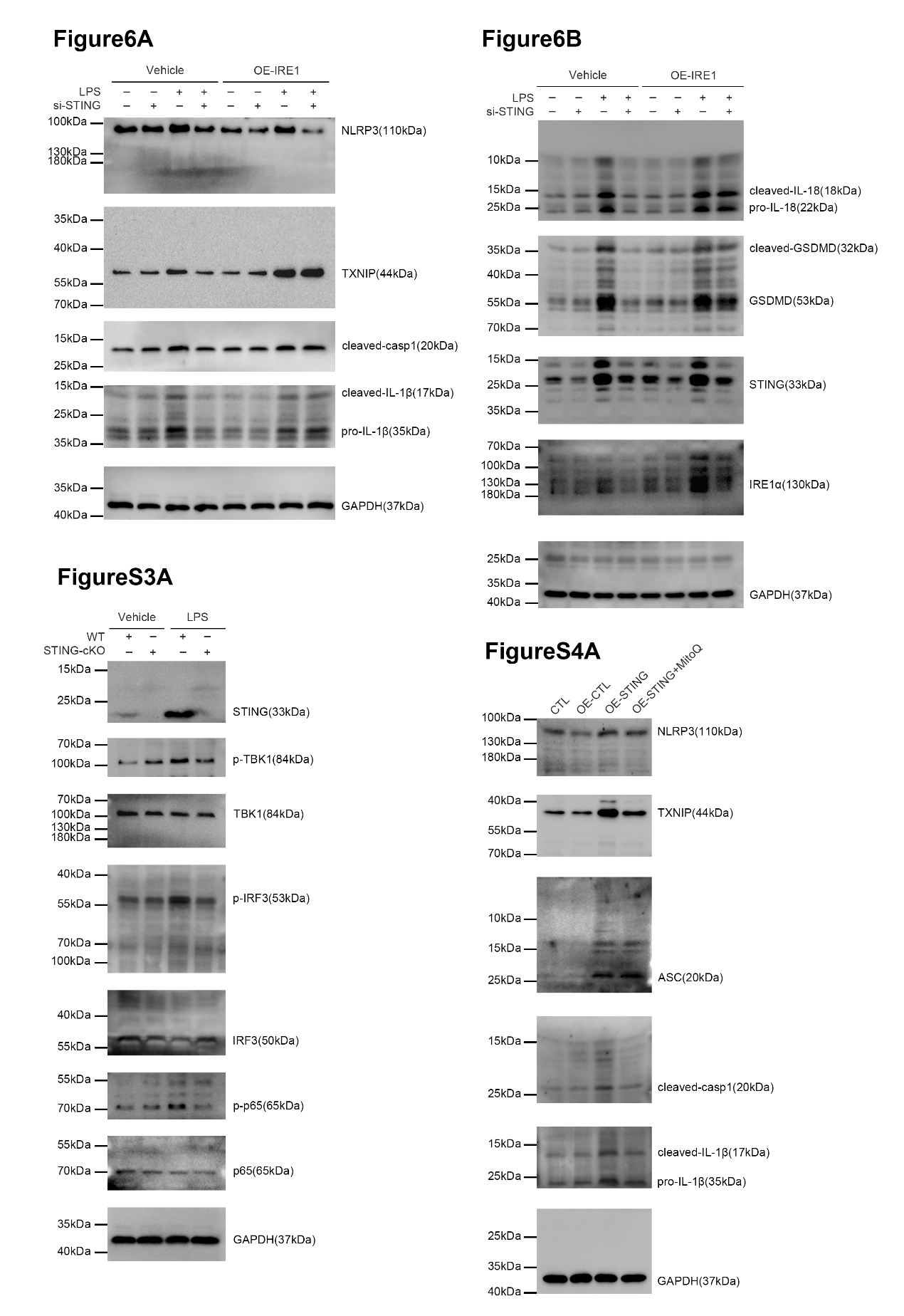


**Original western blots 4**


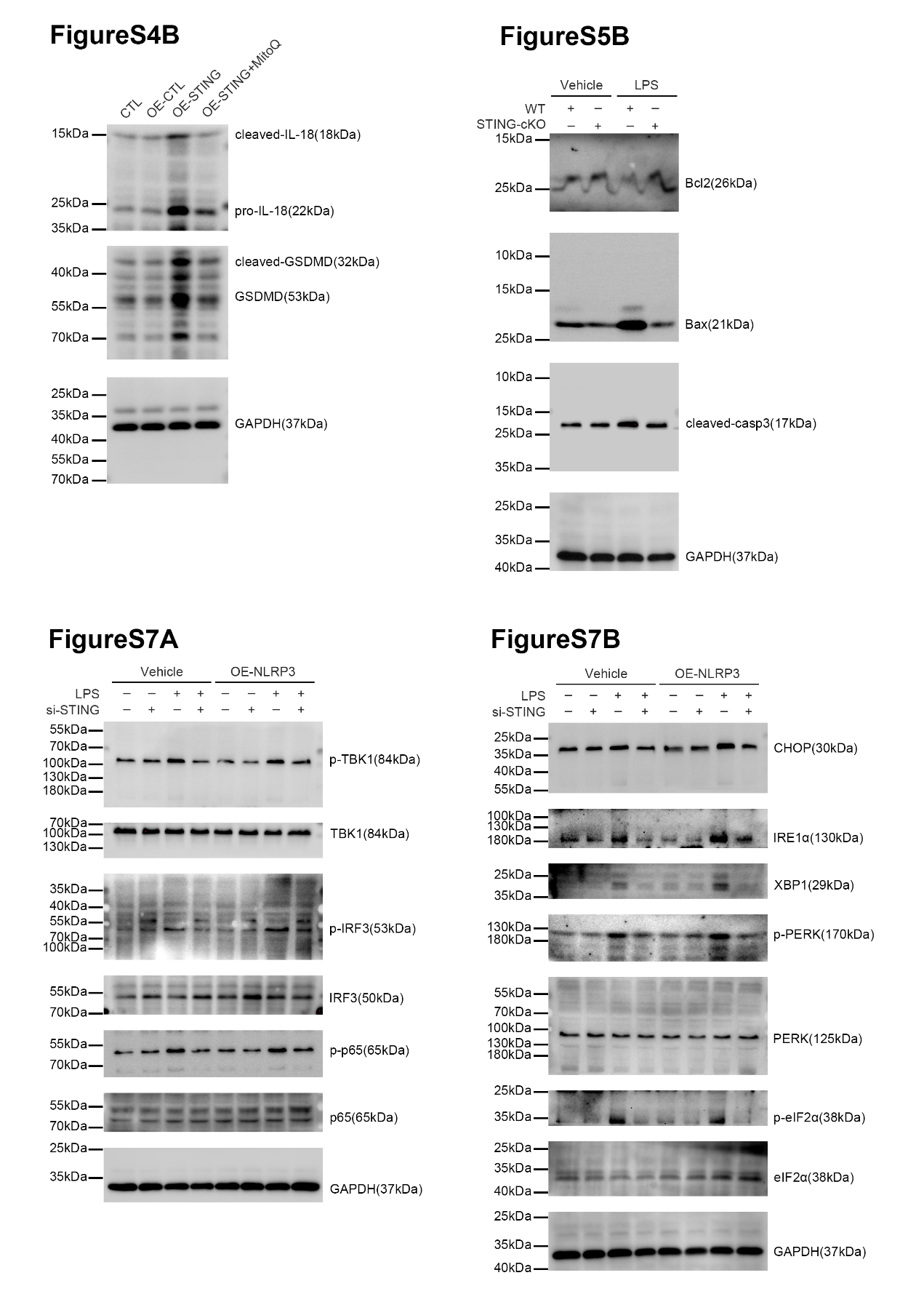

Supplement: Supplementary file 2 — Original Data File [file 41419_2024_6600_MOESM2_ESM.docx]
